# Optical Sensors Technology and Applications

**DOI:** 10.3390/s22207905

**Published:** 2022-10-17

**Authors:** Maria Lepore, Ines Delfino

**Affiliations:** 1 Dipartimento di Medicina Sperimentale, Università della Campania “Luigi Vanvitelli”, I-80138 Napoli, Italy; 2 Dipartimento di Scienze Ecologiche e Biologiche, Università della Tuscia, I-01100 Viterbo, Italy

Optical methods are non-invasive tools, and their use in various fields, including sensing applications, is continuously increasing, which is thanks to the continuous development of innovative low-cost sources and detectors. Together with the use of nanodevices, these new optical technologies enable the development of new sensing schemes and devices characterized by superior working parameters: optical sensors frequently offer very low detection limits, high specificity, and high sensitivity. In addition, they are very versatile and allow the realization of innovative applicative approaches for quantitatively and qualitatively determining the components of analytes of interest in many fields of application, such as in pharmaceutical research, medical diagnostics, environmental monitoring, agriculture, industry, food safety, and security, just to name a few. The present Special Issue aimed to offer an overview of recent advances in optical sensor technology and applications, including source and detection technologies, sensor architectures, sensor performance, and processing approaches and applications.

The polarization of light represents a formidable tool for the development of innovative methods for the control of products and manufacturing processes. Three of the papers in this Special Issue exploit polarization properties for proposing innovative experimental approaches in very different fields [1,2,3]. In Ref. [1], the authors prove that individual measurements of the optical polarization parameters of light scattered by suspended particles are a powerful tool to classify the particulate compositions in seawater. This information plays a pivotal role in ecological research and environmental monitoring. A method based on a dense sampling of polarized light pulses is proposed, and the experimental setup is built. Ref. [2] addresses a completely different field of application of polarization. In fact, this paper presents the implementation of an automatic Stokes dynamic polarimeter to characterize non-biological and biological material samples. Experiments demonstrated the efficiency of the proposed optical array to calculate the Mueller matrix in reflection and transmission mode for different samples. A comparison with similar papers reported in the literature validates the approach proposed by the authors. Ref. [3] is devoted to stress detection of the conical frustum window in deep manned submersibles by using polarization imaging. The authors built a Mueller matrix polarimetry, and the examined samples were similar to the typical conical frustum windows in submersibles. The results evidence that the polarization parameters can characterize the stress transfer process and the elastic–plastic transformation process of the window under different pressurization pressures. This proposed method can offer new possibilities for monitoring the window stress and to further ensure the safety of deep manned submersibles.

Interferometry is an optical technique that allows ingenious approaches in very different experimental situations, as occurs for Refs. [4,5]. In Ref. [4], a three sensors-based Mach–Zehnder interferometer is adopted for developing a non-invasive control method for the burning rate of solid fuel in a model solid propellant rocket motor. The results show that the proposed method allows the non-invasive control of solid fuel burnout both by recording the time of arrival of the combustion front to the sensor and by analyzing the peaks of the signal recorded by the available optical fibers. In Ref. [5], the authors propose the use of a Fizeau interferometer for a self-calibration stitching method for the pitch deviation evaluation of a long-range linear scale. The developed method can represent a significant improvement in the field of optical linear encoders that are widely employed for precision positioning applications, such as semiconductor manufacturing, precision machine tools, and coordinate measuring machines due to their low cost, high resolution, and robustness.

In recent years, the joint use of nanotechnologies and optics has opened new scenarios in the development of sensing schemes. In this framework, the authors of Ref. [6] developed a simple but effective method for the colorimetric detection of antibiotic residues in raw milk by using aptamer-conjugated gold nanoparticles. In fact, the improper use of antibiotics in cattle breeding can cause milk contamination, which has negative effects on the dairy industry and human health. The proposed method could allow the semiquantitative analysis of antibiotic residues in raw milk to be obtained directly from dairy farms. Nanoparticles are also exploited in Ref. [7], in which the authors describe novel flexible plasmonic strain sensors prepared by functionalizing a polymeric transparent substrate with a TiO_2_ thin film containing Au nanoparticles. The use of the localized surface plasmon resonance (LSPR) effect due to the interaction of light with the free electrons of noble metal nanoparticles indicated that the LSPR band of these sensors is sensitive to different applied strains. LSPR bands are mainly modified by the changes in the refractive index of the matrix surrounding the Au nanoparticles when uniaxial strains are applied.

Refs. [8,9] are significant examples of the very numerous and various applications of the vibrational spectroscopies, such as Fourier Transform Infrared (FT-IR) and Raman spectroscopy, that span from basic research, medicine, and biology to agrifood, industry, and many other fields.

In Ref. [8], the authors use FT-IR spectroscopy to discriminate different breast cell lines on glass substrates. They aimed to investigate the possibility of using the FT-IR spectroscopy in the clinical diagnostic field to grow cells by adopting the same substrates that are currently used by cytologists and histopathologists. The reported results contribute to promoting the translation of the FT-IR technique in medical practice, representing a complementary diagnostic tool. Ref. [9] addresses a completely different field of application for Raman spectroscopy. In fact, this paper discusses the possibility of using this spectroscopy for analyzing natural gas by taking advantage of Raman spectroscopy’s high-speed measurement and the potential to monitor all molecular components simultaneously. The adopted contour fit method to derive concentrations from the spectra of mixtures enables reliable results to be obtained, even when a significant change in the composition of the samples is present. The reported results confirm that Raman gas analyzers can operate without frequent calibrations, differently from gas chromatographs.

In Ref. [10], a new criterion for analyzing the resolution and accuracy of the laser speckle correlation method is presented. This would be of great interest and would be relevant for a variety of applications since the laser speckle correlation method is largely applied to obtain information from vibrating objects. The proposed new criterion combines the mean intensity gradient and frequency spectrum and has been verified by using simulated and real speckle patterns with different speckle sizes, densities, and gray contrasts. The authors use rotation and vibration experiments to confirm the effectiveness of the proposed criterion to improve the performance of the laser speckle correlation method.

The use of neural networks allows for the development of a fringe phase extraction method for optical metrology in Ref. [11]. In this framework, a fringe pattern is usually adopted as output from which the desired parameters can be extracted. The authors propose an end-to-end method of fringe phase extraction based on the neural network. The results of simulation and experimental fringe patterns validate the accuracy and the robustness of the proposed method, which is also characterized by easier operation compare to the other methods that are available.

Ref. [12] is a careful review of the conventional and innovative coating thickness methodologies for application to chromium coatings on a ferromagnetic steel substrate and determines their advantages and limitations regarding in-line measurements. The X-ray Fluorescence (XRF) method is the most common in-line coating thickness measurement method employed in the steel packaging industry, but it is expensive and is characterized by health and safety concerns due to its ionizing radiation. Optical methods can represent a valid alternative. The authors discuss three of these methods: optical reflectometry, ellipsometry, and interferometry. The review presents a detailed references section, and the features of the different coating thickness measurement methods are accurately discussed.

Ref. [13] aims to review the most generally used methods for designing and fabricating optical biosensors and sensors for phenolic compounds that are particularly dangerous due to their toxic effects and their ability to persist in the environment for a long period of time. Their danger is also linked to the fact that they are widely used in many industrial processes and agricultural treatments. The basic principles of the most largely used optical detection techniques are presented, and some selected examples of the most interesting applications of these techniques are also discussed. The large number of bibliographic references demonstrate the ever-keen interest in this research area.

The brief discussion of the papers published in the present Special Issue (SI) attests the diversity of the new developments in optical sensors and their wide dissemination in many applied research fields.

Finally, we would like to thank all the authors who have submitted their work to *Sensors*, specifically to this Special Issue, “Optical Sensors Technology and Applications”, all the reviewers for their hard work, and the editors of *Sensors* for their kind and continuous support. All of these efforts made this SI possible. We hope that this SI will help researchers to better understand the state of the art of optical sensors and techniques as well as their wide range of applications. We hope that the 13 published papers will also help the researchers working in the field to disclose future perspectives.
